# Effect of malaria preventive education on the use of long-lasting insecticidal nets among pregnant females in a Teaching Hospital in Osun state, south-west Nigeria

**DOI:** 10.1016/j.parepi.2020.e00182

**Published:** 2020-09-19

**Authors:** Omowonuola O. Sonibare, Ibrahim S. Bello, Samuel A. Olowookere, Olabode Shabi, Niyi O. Makinde

**Affiliations:** a Department of Family Medicine, Obafemi Awolowo University Teaching Hospitals Complex, Ile-Ife, Nigeria; b Department of Community Health, College of Health Sciences, Obafemi Awolowo University, Ile-Ife, Nigeria; c Federal Medical Centre, Ido-Ekiti, Nigeria; d Department of Obstetrics and Gynaecology, College of Health Sciences, Obafemi Awolowo University, Ile-Ife, Nigeria

**Keywords:** Malaria preventive education, Pregnant females, Long-lasting insecticidal net, Antenatal clinic

## Abstract

Background: Malaria in pregnancy is a major public health problem in Nigeria. Long-Lasting Insecticidal Nets (LLINs) have been advocated as an effective tool against malaria transmission. However, success of this intervention largely depends on the knowledge and practices regarding malaria and its prevention. Unfortunately, few studies have been done on effect of malaria preventive education on use of LLIN in pregnancy.

Objective: To assess the knowledge of malaria and determine the effect of malaria preventive education on the use of LLINs among pregnant females in a Teaching Hospital in Osun state.

Method: It was a one group pre-test post-test quasi - experimental hospital based study involving pregnant females attending Ante-Natal Clinic (ANC) of the Obafemi Awolowo University Teaching Hospitals Complex (OAUTHC). A total of 200 respondents were recruited for this study by 2-stage sampling technique. Data was collected using a pretested questionnaire to elicit information on socio-demographic characteristics, use of LLIN in pregnancy, knowledge of malaria and its preventive measures. The data collected was analysed using descriptive and inferential statistics. The descriptive statistics comprised frequency, percentage, means and standard deviations. Bivariate analysis comprised Chi-square test on knowledge of malaria before and after intervention while correlation test assessed strength of relationship between knowledge of malaria preventive education and use of LLINs before and after intervention. Multivariate analysis determined the predictors of LLINs use. Analytical statistics of cross tabulation was conducted considering a *p* < 0.05 to be statistically significant.

Results: There was an increase in the scores of knowledge on malaria transmission after the intervention and this was statistically significant (χ^2^ = 8.862, *p* < 0.01). Similarly, the scores of knowledge on malaria prevention increased after the intervention and this was statistically significant (χ^2^ = 10.023, *p* < 0.01). Respondents' age, marital status and gravidity were predictors of LLINs use. Biserial correlation showed a statistically positive relationship between knowledge of malaria preventive education and use of LLINs after intervention (*r* = 0.036, *p* < 0.01).

Conclusion: The use of malaria preventive education was found to be effective in increasing the use of LLIN in this study. These findings highlight a need for educational intervention in implementation of LLINs. There is therefore a need to strengthen the policy of malaria prevention education as an integral component with distribution of free LLIN in health care setting to enhance its utilization.

## 1 Introduction

Malaria is a vector borne disease that poses an enormous burden to the world's population. Sub-Saharan Africa (SSA) has the largest burden of malaria disease, with over 90% of the world's malaria-related deaths occurring in this region (World Health Organization (WHO), 2012). Malaria is transmitted throughout Nigeria with 97% of the population at risk of the infection (Dawaki et al., 2016). It directly accounts for about 11% of all maternal deaths in Nigeria mainly by being a leading cause of anaemia in pregnancy (Ankomah et al., 2012). Malaria is transmitted through the bite of an infected female *Anopheles* mosquito with higher risk occurring in population groups such as children less than 5 years and pregnant women (World Health Organization (WHO), 2019). In Nigeria, pregnant women are mostly infected with *Plasmodium falciparum*, the most virulent *Plasmodium* with serious health consequences including anaemia, stillbirth, and premature delivery (Onoka et al., 2012).

Malaria is endemic throughout Nigeria (Yakubu et al., 2019) with a seasonal transmission pattern, the peak transmission of which is during the rainy season (April to October) followed by the dry season (November to March). Its endemicity is responsible for annual economic loss of N132 billion according to the National Malaria Control Programme (Auta, 2012). It is also responsible for 60% of out-patient visits to health facilities and 30% of hospitalization. In addition, at least 50% of the population has at least one episode of malaria annually (Salihu and Sanni, 2013). Also, parasite prevalence in pregnant women in Nigeria could be as high as 60–70% (Onoka et al., 2012), and being a killer disease, it kills poor pregnant women and children hence causing an increase in maternal and child mortality in Nigeria (Chukwuocha et al., 2012). This explains why national efforts to reduce the high maternal and infant mortality place high premium on effective control of malaria in pregnancy (Takem and D'Alessandro, 2013). The current World Health Organization (WHO) strategic approach to prevention and control of malaria in pregnancy in areas of stable *Plasmodium falciparum* transmission is three-pronged that include Intermittent Preventive Treatment in pregnancy (IPTp), Long-Lasting Insecticidal Net (LLIN) use and prompt case management of malaria (World Health Organization (WHO), 2014). Prevention of malaria in pregnancy is a major public health challenge and a priority for the Roll Back Malaria (RBM) partnership (Onwujekwe et al., 2012). The current prevention tools such as use of LLINs and IPTp though available, face a number of important limitations to their uptake in Nigeria. The RBM African Summit held in Abuja, Nigeria made a commitment known as the Abuja Declaration, where it agreed that 60% of pregnant women in malaria endemic areas should have access to effective treatment and prevention of malaria with IPTp and Insecticide Treated Net (ITN) by 2005. Later the target was increased to reach 80% of pregnant women by 2010 (Akoria and Arhuidese, 2014). LLINs had been shown to be beneficial and should be included in strategies being promoted to reduce the adverse effects of malaria in pregnant women in endemic areas of the world (Soleimanmi-Ahmadia et al., 2012).

The efficacy and cost-effectiveness of LLINs in reducing malaria related morbidity and mortality had led to massive efforts to distribute millions of free or highly subsidized LLINs to vulnerable population in SSA (Ntuku et al., 2017). Despite the current initiative developed to address the public health challenges malaria poses, the problem still persists. This simply implies that research should be directed towards personal level characteristics of persons defined to be at high risk of malaria transmission. It is now well established that health behaviour has links to health outcomes and these links in turn are dependent on factors associated with cognitive processes of reasoning and health literacy, quality of health care services, available health related information and decision making process at the individual level (Atulolmah et al., 2014).

Health literacy acquired through appropriate health education provides the necessary action-stimulating impetus to engage in preventive health (Atulomah and Atulomah, 2012). WHO defines health education as consciously constructed opportunities for learning involving some form of communication designed to improve health literacy, including improving knowledge and developing life skills which are conducive to individual and community health (World Health Organization, 2012). The purpose of health education is to positively influence the health behaviour of individuals and communities as well as the living and working conditions that influence their health (Coalition of National Health Education Organizations, 2009). Disease prevention covers measures not only to prevent the occurrence of disease such as risk factor reduction, but also to arrest its progress and reduce its consequences once established (World Health Organization, 2012). By focusing on prevention, health education reduces the costs (both financial and human) that individuals, employers, families, companies, medical facilities, communities, the state and the nation would spend on medical treatment (Coalition of National Health Education Organizations, 2009). Malaria Preventive Education focuses on prevention strategies to reduce the risk of developing malaria and associated morbidities by empowering behaviour change and actions through increased knowledge. Examples of education strategies include courses, trainings and support groups (Rural Health Information Hub, 2020). Preventive education should be designed to fill in knowledge gaps, overcome negative perceptions and provide motivation. Proven effective options to reduce morbidity and mortality include malaria prevention through reduction of human-vector contact, especially with the use of LLINs (World Health Organization (WHO), 2007).

Many pregnant women are not aware of the risk and consequences of infectious diseases and thus not practicing preventive strategies (Cannon et al., 2012). Women are susceptible to malaria infection due to changes in the immune system during pregnancy and the presence of placenta as parasite binding sites (Centers for Disease Control and Prevention, 2018; World Health Organization (WHO), 2017). In SSA, some studies done have shown that women's knowledge on malaria is low (Omaka-Omari and Nwimol, 2015; Yaya et al., 2017; Tayseir et al., 2017; Akaba et al., 2013; Obol et al., 2011). Having a good knowledge on malaria cause, mode of transmission, sign and symptom, complication of malaria in pregnancy and prevention of malaria leads to use of malaria prevention strategies (Fuge et al., 2015; Emmanuela et al., 2011; Tamirat et al., 2016; Ayiisi, 2017). Studies done in Ethiopia (Goshu and Yitayew, 2019), Burkina Faso (Yaya et al., 2017), and Sudan (Tayseir et al., 2017) showed that 73.2%, 56.1% and 55.9% of respondents had good knowledge on malaria respectively. A study done in Nigeria revealed that although 96.2% of the respondents were aware malaria was caused by infected mosquito bite, there was poor knowledge of its complications in both mother and foetus (Okafor et al., 2019). In Cameroon, a study assessing the knowledge of mode of transmission and prevention of malaria among pregnant women showed that though 64% of the respondents had knowledge on mode of transmission, majority don't have effective knowledge on malaria prevention (Nkfusai et al., 2019). Knowledge is a crucial element in health improvement (Iriemenam et al., 2011). To improve the effectiveness of malaria control interventions, education of a disease-burdened group such as pregnant women is essential (Ouattara et al., 2011). There is a need to deepen their knowledge on ways of malaria prevention for attainment of self-reliance (Iriemenam et al., 2011).

It is noteworthy that education intervention had been observed by various studies as a valuable tool in malaria prevention and control in SSA (Protopopoff et al., 2007). Results of a recent study done by Kumar et al. in Pakistan suggested that educational intervention is an effective means of improving malaria knowledge and LLINs use among pregnant women (Kumar et al., 2020). Similarly, a study done among nursing mothers by Amoran in Ogun state, south-west, Nigeria showed that education intervention remain effective in influencing behaviour change and improving knowledge of malaria and benefits of LLIN use (Amoran, 2013). Another study done by Ahmadi et al in Iran found a significant increase in LLIN use among households due to an education intervention (Ahmadi et al., 2012). Thus, understanding malaria preventive measures by pregnant mothers attending Ante-Natal Clinic (ANC) is an essential element in malaria control. This study therefore assessed the effect of malaria preventive education on the use of long-lasting insecticidal nets among pregnant females in Obafemi Awolowo University Teaching Hospitals Complex (OAUTHC), Osun state, south-west, Nigeria.

## 2 Methodology

### 2.1 Study site

**Fig. 1:**
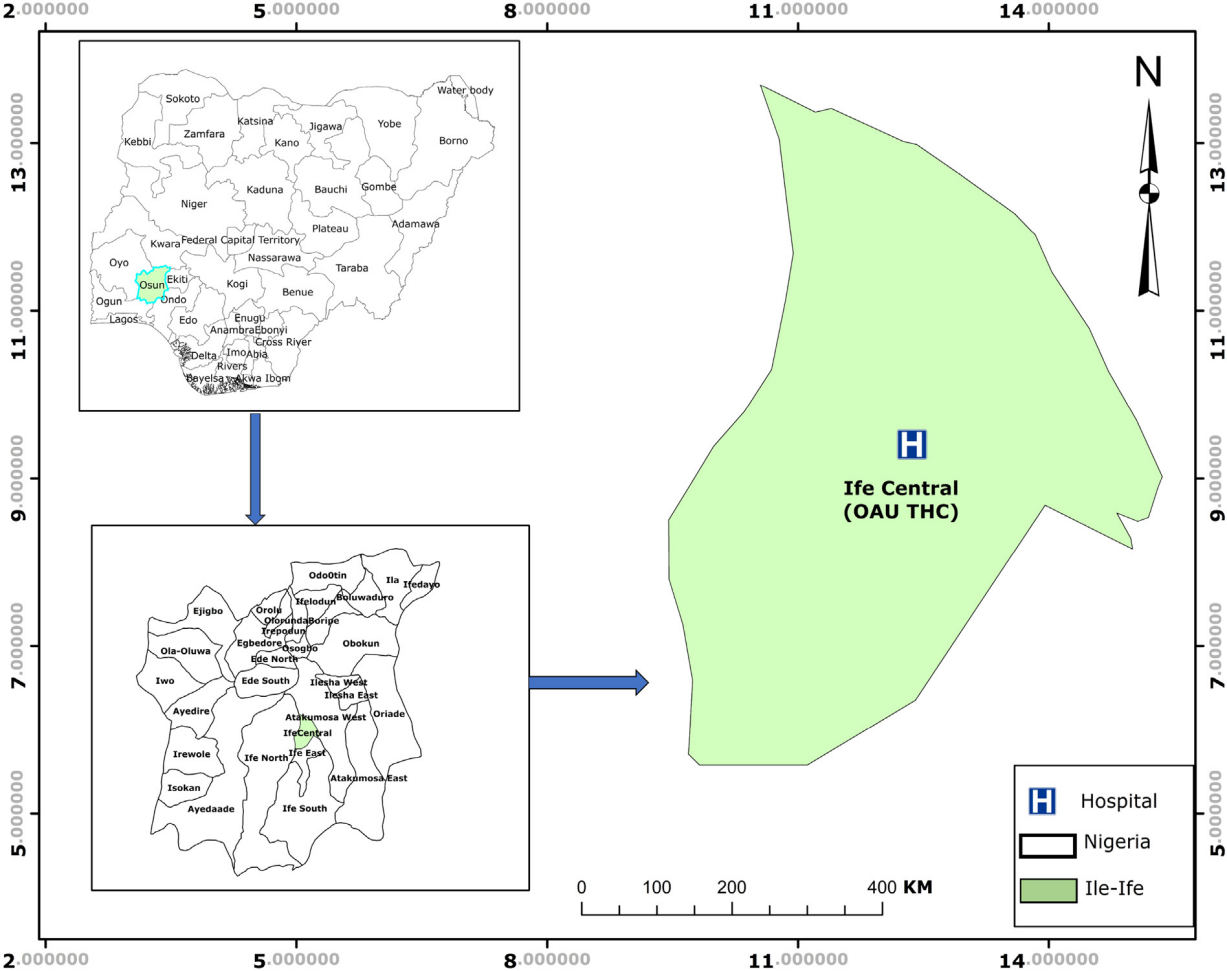
The Study Location.

The study was carried out in Ile-Ife, an ancient town in south western Nigeria located between latitudes 7°28′N and 7°45′N and longitudes 4°30′E and 4°34′E (Fig. 1). Ile-Ife has a population of 501,952 (Ajala and Olayiwola, 2013) and is surrounded by rural settlements where agriculture is the chief occupation of the inhabitants. It is also a highly commercialized city with educational and health institutions at primary, secondary and tertiary levels including a University Teaching Hospital. The humidity is high in Ile-Ife and its environs and like most other parts of southern Nigeria, the climate is tropical with two seasons: the rainy and dry seasons.

### 2.2 Study design

This study was a quasi - experimental hospital based study. One group pre-test post-test design in which pregnant females who presented for ANC visits in OAUTHC were interviewed using structured questionnaires in line with the objective of the study. The study took place from July to December 2015.

### 2.3 Study population

Inclusion criteria: Newly booked pregnant females aged 15 to 49 years in their first or second trimester of pregnancy.

Exclusion criteria: All acutely ill pregnant females were excluded in order to receive urgent care.

### 2.4 Sample size determination

The minimum sample size was calculated using the Leslie Kish formula;

$$n=Z^{2}\mathrm{pq}/d^{2}$$

where,n = minimum sample size.Z = standard normal deviate set at 1.96 which corresponds to the 95% confidence level.d = degree of accuracy desired (set confidence interval) at 0.05.p = Estimate of prevalence of malaria in pregnancy. An adequate and reliable minimum sample size was determined using the prevalence of malaria in pregnancy derived from a study carried out in south western Nigeria (Osogbo), which was 13% (Adeleke et al., 2013).

q=1−p

$$n=1.96\times 1.96\times 0.13\times \left(1-0.13\right)/0.05\times 0.05$$

Thus, *n* = 174.

Minimum sample size was 174.

Adding 10% attrition, the total number of respondents to be recruited was 191. The sample size was however rounded up to 200.

### 2.5 Sampling technique

Systematic and simple random sampling methods were used for the study. On each booking clinic day, usually 50–60 pregnant females are booked at the nursing station and a list of 40 pregnant females that fulfilled the inclusion criteria was directed to the researchers. Forty (40) respondents were enlisted for a period of 10 weeks giving a total of 400 respondents over the study period while the sample size was 200. The sampling interval, k = 400/200 = 2. Thus, every 2nd consenting respondent represented the sample interval for the study. The first respondent was selected by simple random sampling technique (by balloting), and one consenting respondent was recruited. Subsequent respondents were selected using the sampling interval until the desired sample size was attained.

### 2.6 Data collection tools

A pretested structured interviewer administered questionnaire was used to collect the data. The questionnaire had three sections which assessed the respondents' socio-demographic characteristics, use of LLIN in pregnancy and knowledge of malaria and its preventive measures. Content and face validation of the instrument was done by a panel of expert (Obstetrics and Gynaecology specialist, Public Health Physician and Family Physician) in the field of the study. The questionnaire was pretested among pregnant females attending Urban Comprehensive Health Centre, Eleyele, Ile-Ife and found suitable for the study. The Cronbach's alpha reliability coefficient was calculated to be 0.83. The instrument was translated into the local language (Yoruba) and back translated to English Language. Three research assistants were recruited and trained for 2 days to assist with data collection.

#### 2.6.1 Respondent's recruitment

**Fig. 2:**
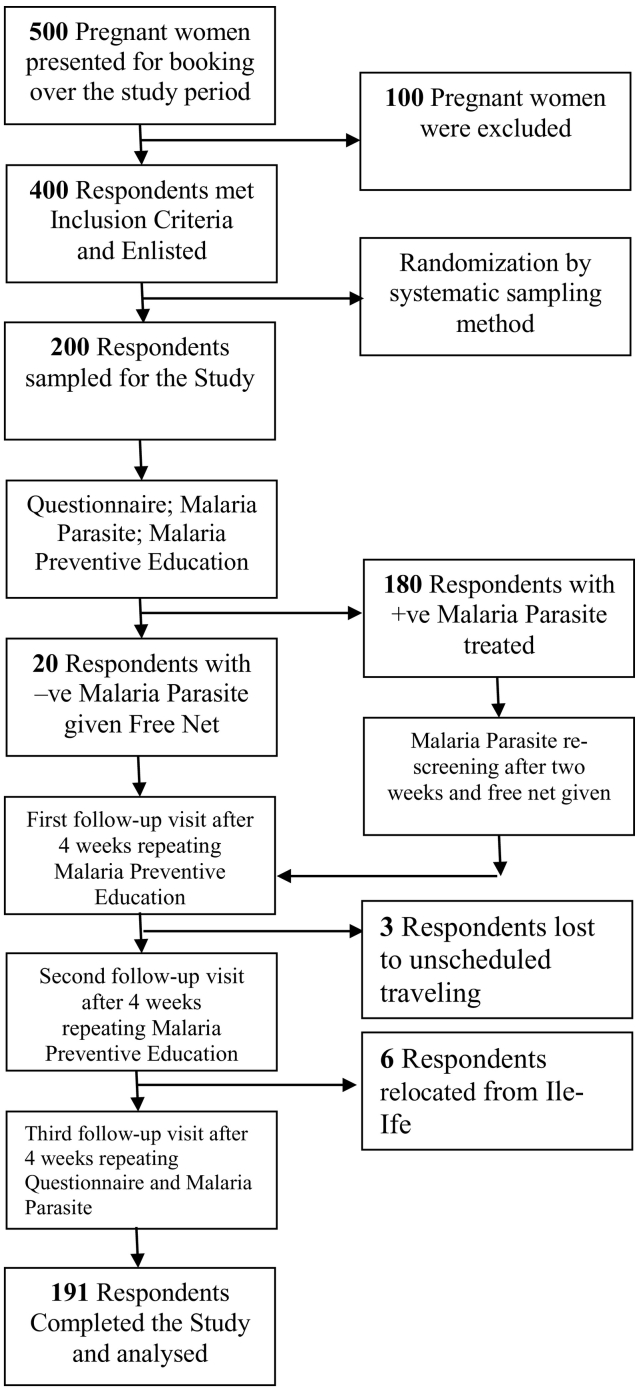
The Study Flow Chart.

During the 10 weeks recruitment period, 500 respondents presented for booking out of which 400 met the inclusion criteria and gave consent to participate in the study. Systematic sampling method was applied on the recruited 400 respondents until the sample of 200 respondents was reached. During the period of the study 9 respondents dropped out: 3 respondents had unscheduled travelling after the first follow up visit while 6 respondents relocated outside the state after the second follow up visit. Therefore 191 respondents completed the study and their responses were included in the data analysis. The study flow chart is shown in Fig. 2.

### 2.7 Method of data collection

At pre-intervention stage, the questionnaire was administered and the respondents were screened for malaria parasite using microscopy. Respondents that were malaria parasite positive were treated free following the National guideline for treatment of uncomplicated malaria in pregnancy. All the respondents continued their routine antenatal packages which are; health education on prevalent health issues in pregnancy, haematinics, IPTp by directly observed treatment (D.O.T) and tetanus toxoid vaccine.

At intervention stage, on an interpersonal basis, each respondent was educated using a structured malaria preventive educational programme along with a graphic description of malaria life cycle and transmission. Free LLINs were made available to each respondent with education on installation and use of the nets. Each respondent had 3 visits on a monthly basis and during their follow-up visit, the malaria preventive education was repeated and the respondents were encouraged to use their nets every night.

At post-intervention stage, the same questionnaire that was administered at pre-intervention was again administered to all the respondents at their 12th week from the time of enrolment.

### 2.8 Data processing and analysis

Data collected were analysed using the Statistical Package for Service Solution (SPSS version 20). Frequency/percentages for categorical variables were generated for socio-demographic characteristics of the respondents. Chi square test was used to determine knowledge of malaria before and after malaria preventive education. Correlational test was used to assess the strength of the relationship between knowledge of malaria preventive education and use of LLINs before and after the intervention. Multivariate analysis (binary logistic regression) was used to identify factors influencing LLINs use. The relationship between variables tested using Chi square was at 5% confidence level.

### 2.9 Determination of use of LLIN by the respondents

LLIN use was measured in three categories: non-use, occasional use and consistent use. LLIN use by the respondents was re-categorized into two such that occasional users and non-users were collapsed into one group, while consistent users remained as the second group.

### 2.10 Determination of knowledge of malaria and the preventive measures of the respondents

The knowledge of malaria was assessed using a 12-item multiple-choice question. The questions assessed the knowledge of malaria transmission, treatment and prevention. Each response was scored as ‘yes’ or ‘no’. Knowledge was assessed by giving 1 to correct answer and 0 to the wrong answer. An overall knowledge score was calculated by adding up the scores for each respondent across the 12 questions. The mean knowledge score for all the respondents was 26.57 (SD = 2.38). Categorization into good or poor knowledge was by the mean scores of the respondents. Scores above the mean were categorized as good knowledge while scores below the mean were categorized as poor knowledge.

### 2.11 Ethical approval and consent to participate

Ethical clearance was obtained from the Ethical Committee of the Obafemi Awolowo University Teaching Hospitals Complex, Ile-Ife, Nigeria. A clearance certificate was issued with registration number: IRB/IEC/0004553. Written informed consent was obtained from the respondents, parents or guardian in the form of signatures or thumb impressions.

## 3 Results

### 3.1 Socio-demographic characteristics of the study respondents

The sample consisted of 200 respondents who started the study with 191 completing it (Table 1). This gave a response rate of 95.5%. Most (69.9%) of the women were aged 25–34 years, 89.0% were in their second trimester and 70.2% of the women were multigravidae. Majority (89.5%) were of Yoruba tribe and about 78.5% had tertiary education while 72.8% were of high socio-economic class. Majority (96.3%) were married while 92.1% lived in households with less than 5 persons.

**Table 1:** Frequency distribution of respondents by socio demographic characteristics (*N* = 191).

Socio-demographic characteristics	Frequency (*n*)	Percentage (%)
Age group in years		
15–24	24	12.6
25–34	133	69.6
35–44	30	15.7
45–49	4	2.1

Ethnicity		
Hausa	1	0.5
Igbo	12	6.3
Yoruba	171	89.5
Itsekiri	7	3.7

Marital status		
Single	7	3.7
Married	184	96.3

Education		
Primary	7	3.7
Secondary	34	17.8
Tertiary	150	78.5

Occupation		
Civil servants	65	34.0
Trader	69	36.1
Artisan	19	9.9
Unemployed	38	19.9

Residence		
Rural	52	27.2
Urban	139	72.8

Religion		
Christianity	158	82.7
Islam	33	17.3

Socioeconomic status		
High	139	72.8
Middle	36	18.8
Low	16	8.4

Household size		
1–5	176	92.1
6–10	15	7.9

Gravidity		
Primigravidae	57	29.8
Multigravidae	134	70.2

Trimester		
1st	21	11.0
2nd	170	89.0

### 3.2 Respondents' knowledge on malaria and its preventive measures

Table 2 shows the respondents' knowledge on malaria and its preventive measures. Before malaria preventive education, the proportion of respondents who had good knowledge (18.3%) on malaria transmission were lower than the proportion (30.9%) at post-intervention (χ^2^ = 8.862, *p* = 0.003). Also, the proportion of respondents who had good knowledge (11.5%) on malaria prevention before intervention were lower than the proportion (31.9%) at post-intervention (χ^2^ = 10.023, *p* = 0.001). It was also observed that 35.1% of the respondents had poor knowledge on treatment of malaria before intervention compared to 9.9% at post intervention (χ^2^ = 16.217, *p* = 0.000).

**Table 2:** Two-sample proportion chi-square for knowledge before and after intervention.

	After		Total	df	Pearson chi-square	
	Poor knowledge	Good knowledge			χ^2^	*p*-Value
Transmission: before						
Poor knowledge	57	40	97			
Good knowledge	35	59	94	1	8.862^a^	0.003
Total	92	99	191			

Consequence: before						
Poor knowledge	45	87	132	1	0.183	0.669
Good knowledge	22	37	59			
Total	67	124	191			

Symptom: before						
Poor knowledge	60	70	130	1	0.781	0.377
Good knowledge	24	37	61			
Total	84	107	191			

Treatment: before						
Poor knowledge	67	19	86	1	16.217^a^	0.000
Good knowledge	52	53	105			
Total	119	72	191			

Prevention: before						
Poor knowledge	53	55	108	1	10.023^a^	0.001
Good knowledge	22	61	83			
Total	75	116	191			

a Indicates significant at 1%.

### 3.3 Effect of malaria preventive education on use of LLINs before and after intervention

As presented in Table 3, respondents who were 25 years and above were 87% less likely to use LLINs before and after intervention than those less than 25 years (OR = 0.128, 95% CI = 0.027–0.602). Respondents who were married are 11 times more likely to use LLINs before and after intervention than those who are single (OR = 11.686, 95% CI = 1.111–122.898). Also, respondents who were primigravidae were 83% less likely to use LLINs before and after intervention than multigravidae (OR = 0.171, 95% CI = 0.034–0.865). There was an increase in the overall proportion of respondents who used LLINs from 11.0% before malaria preventive education to 83.2% after the intervention indicating a positive effect of malaria preventive education on LLINs use (OR = 2.283, 95% CI = 0.599–8.692). Also, there was a statistically positive relationship between knowledge of malaria preventive education and use of LLINs at post-intervention compared to baseline (*r* = 0.036, *p* < 0.01) as presented in Table 4.

**Table 3:** Effect of malaria preventive education on use of LLINs before and after intervention.

Variables	LLINs	Before	After	B	df	*p*-Value	Exp(B)	95% C·I for EXP(B)	
								Lower	Upper
Overall	Use	21 (11%)	159 (83.2%)	0.825	1	0.226	2.283	0.599	8.692
	Non-use	170 (89%)	32 (16.8%)						

Age groups									
<25	Use	3 (12.5%)	21 (87.5%)	−2.052	1	0.009	0.128	0.027	0.602
	Non-use	21 (87.5%)	3 (12.5%)						
≥25	Use	18 (10.8%)	138 (82.6%)						
	Non-use	149 (89.2%)	29 (17.4%)						

Residence									
Rural	Use	5 (9.6%)	43 (82.7%)	−0.775	1	0.276	0.461	0.114	1.86
	Non-use	47 (90.4%)	9 (17.3%)						
Urban	Use	16 (11.5%)	116 (83.5%)						
	Non-use	123 (88.5%)	23 (16.5%)						

Education									
Secondary	use	5 (12.2%)	32 (78%)	0.414	1	0.662	1.513	0.236	9.691
	Non-use	36 (87.8%)	9 (22%)						
Tertiary	Use	16 (10.7%)	127 (84.7%)						
	Non-use	134 (89.3%)	23 (15.3%)						

Socioeconomic status									
High	Use	15 (10.8%)	117 (84.2%)	0.994	1	0.392	2.703	0.277	26.332
	Non-use	124 (89.2%)	22 (15.8%)						
Middle/Low	Use	6 (11.5%)	42 (80.8%)						
	Non-use	46 (88.5%)	10 (19.2%)						

Ethnicity									
Yoruba	Use	18 (10.9%)	133 (80.6%)	−0.839	1	0.461	0.432	0.046	4.026
	Non-use	147 (89.1%)	32 (19.4%)						
Others	Use	3 (11.5%)	18 (69.2%)						
	Non-use	23 (88.5%)	8 (30.8%)						

Marital status									
Single	Use	0 (0.0%)	5 (71.4%)	2.458	1	0.041	11.686	1.111	122.898
	Non-use	7 (100%)	2 (28.6%)						
Married	Use	21 (11.4%)	146 (79.3%)						
	Non-use	163 (88.6%)	38 (20.7%)						

Household size									
≤5	Use	18 (11.5%)	123 (78.3%)	−0.44	1	0.613	0.644	0.118	3.532
	Non-use	139 (88.5%)	34 (21.7%)						
>5	Use	3 (8.8%)	28 (82.4%)						
	Non-use	31 (91.2%)	6 (17.6%)						

Gravidity									
Primigravidae	Use	3 (5.3%)	42 (73.7%)	−1.768	1	0.033	0.171	0.034	0.865
	Non-use	54 (94.7%)	15 (26.3%)						
Multigravidae	Use	18 (13.4%)	109 (81.3%)						
	Non-use	116 (86.6%)	25 (18.7%)						

Occupation									
Civil servants	Use	10 (15.9%)	50 (79.4%)	−0.093	1	0.883	0.911	0.264	3.144
	Non-use	53 (84.1%)	13 (20.6%)						
Traders/artisans	Use	10(12.0%)	70 (84.3%)						
	Non-use	73 (88.0%)	13 (15.7%)						
Unemployed	Use	1 (2.2%)	31 (68.9%)						
	Non-use	44 (97.8%)	14 (31.1%)						

Trimester									
1st trimester	Use	3 (14.3%)	16 (76.2%)	1.028	1	0.124	2.795	0.755	10.349
	Non-Use	18 (85.7%)	5 (23.8%)						
2nd trimester	Use	18 (10.6%)	135 (79.4%)						
	Non-use	152 (89.4%)	35 (20.6%)						

Religion									
Christianity	Use	14 (8.9%)	127 (80.4%)	0.419	1	0.57	1.52	0.359	6.44
	Non-Use	144 (91.1%)	31 (19.6%)						
Islam	Use	7 (21.2%)	24 (72.7%)						
	Non-use	26 (78.8%)	9 (27.3%)						
Constant				−1.319	1	0.732	0.267

**Table 4:** Biserial correlation between knowledge of malaria preventive education and use of LLINs before and after intervention.

Variables	Between Use of LLINs and Knowledge: Before	
	Use of LLINs	Knowledge: before
Use of LLINs	1	
Knowledge: before	−0.034	1


Variables	Between use of LLINs and Knowledge: after	
	Use of LLINs	Knowledge: After
Use of LLINs	1	
Knowledge: after	0.036^a^	1

a Correlation is significant at the 0.01 level (2-tailed).

## 4 Discussion

Malaria in pregnancy is a major public health concern in Nigeria and other SSA countries. It has many deleterious effects on both the mother and foetus underscoring the significance of making available to these groups of people adequate and effective protection (Afolabi et al., 2014).

### 4.1 Knowledge of malaria

This study revealed evidence of knowledge gaps about malaria transmission by majority of the respondents who reported that malaria is transmitted through cold weather, excessive sunlight and eating of cold food. Studies in Nigeria and parts of Africa have also reported spurious causes of malaria such as staying for long in the sun and drinking bad water among other misconceptions on malaria transmission (Obol et al., 2011; Olayemi et al., 2012; Shimaponda-Mataa et al., 2017; Aju-Ameh et al., 2016). These misconceptions could adversely affect preventive behaviour and emphasizes the need for effective malaria preventive education programme to improve the level of knowledge in the study population which is critical for malaria prevention and control using LLINs. Improvement in knowledge of malaria transmission and prevention is essential for promoting proper use of LLINs (Baume and Marin, 2007).

### 4.2 Effect of malaria preventive education on the use of LLINs among the respondents

Another important finding of this study was an increase in the use of LLINs following malaria preventive education. Similarly, there are studies that have shown increase in the use of LLINs when respondents received educational activity (Kumar et al., 2020; Ahmadi et al., 2012; Envuladu et al., 2012). Some factors that affect LLINs utilization in pregnancies include educational status, age groups, employment status and income level (Nkoka et al., 2018). In our study, factors such as age, marital status and gravidity were found to have a significant positive predictive factor for the use of LLINs among the respondents. Similar to our findings, a study done in Cameroon found gravidity as a factor influencing LLINs use (Fokam et al., 2016) while another study done in southern Rwanda found that the respondents' age influenced LLINs use (Habimana et al., 2020). However, studies done in endemic counties recognised positive relation between educational status and improved use of LLINs (Ahmadi et al., 2012; Biswas et al., 2010). Also, some other studies showed no significant association between identified factors and LLINs use. The inconsistencies in studies might be due to environmental factors (Hills et al., 2013). Malaria preventive education programme with the free distribution and delivery of LLINs at the hospital could explain the increase in motivation for LLINs use among the respondents.

### 4.3 Strength and limitation of the study

The study was conducted in malaria endemic area where malaria had public health importance using raw data. Though a facility based study with limited funds available for execution of the study, it still serves as a re-awakening for stakeholders and policy makers as there are global recent calls for renewed attention on malaria in pregnancy (MIP) as part of efforts to achieve the Sustainable Development Goal (SDG) 3. LLIN use was based on self-report and therefore liable to information bias such as over reporting and there was lack of true randomization, despite these shortcomings, the study provides relevant information in the context of MIP in OAUTHC.

### 4.4 Conclusion

In this study the use of malaria preventive education was found to be effective in increasing the use of LLIN. These finding highlight a need for educational intervention in implementation of LLINs. There is therefore a need to strengthen the policy of malaria prevention education as an integral component with distribution of free LLIN in health care setting to enhance its utilization.

## List of abbreviations

LLIN(s): Long-Lasting Insecticidal Net(s)
ITN: Insecticide Treated Net
SSA: Sub Sahara Africa;
IPTp: Intermittent Preventive Treatment in pregnancy
RBM: Roll Back Malaria
ANC: Ante-Natal Clinic
DOT: Directly Observed Treatment
OAUTHC: Obafemi Awolowo University Teaching Hospitals Complex
WHO: World Health Organization
SDG: Sustainable Development Goal
MIP: Malaria in Pregnancy

## Declaration of Competing Interest

None.

## Appendix A Supplementary data

Supplementary material Image 1
