# Supplementary material for: Effect of malaria preventive education on the use of long-lasting insecticidal nets among pregnant females in a Teaching Hospital in Osun state, south-west Nigeria
Source: Parasite Epidemiol Control. 2020 Sep 19;11:e00182. doi: 10.1016/j.parepi.2020.e00182 (PMC7519353; doi:10.1016/j.parepi.2020.e00182)
Supplement: Supplementary file 1 — Supplementary material [file mmc1.docx]

**SUPPLEMENTARY FILE**

**RESEARCH QUESTIONNAIRE ON EFFECT OF LONG LASTING INSECTICIDE NETS AND MALARIA PREVENTIVE EDUCATION ON MALARIA PARASITAEMIA AMONG PREGNANT WOMEN ATTENDING ANTENATAL CLINIC OF OBAFEMI AWOLOWO UNIVERSITY TEACHING HOSPITALS COMPLEX, ILE-IFE**

Dear respondents, this is a study on the effect of long lasting insecticide nets and malaria preventive education on malaria parasitaemia among pregnant women attending antenatal clinic of ObafemiAwolowo University Teaching Hospitals complex, Ile-Ife. This will help us serve you better and your cooperation is needed to answer the questions truthfully. The information you provide will be treated with the highest level of confidentiality and will take few minutes. Thanks for your cooperation.

**DATE OF INTERVIEW: day [ ][ ] month [ ][ ] year [ ][ ][ ][ ]**

**Serial No:________________________ Follow up date: 1^st^ Contact ………..**

**2^nd^ Contact ……….**

**3^rd^ Contact/12wks…**

| **SECTION A SOCIO-DEMOGRAPHIC DATA** | | | | |
| --- | --- | --- | --- | --- |
| ***If you don’t mind, I would like to start by asking you some questions about yourself.*** | | |  |  |
| 01 | Hospital No | ……………………………………….. |  |  |
| 02 | How old were you on your last birthday? | Age (in years) .[ ] ] |  |  |
| 03 | Residential Address | …….…………………………… |  |  |
| 04 | Mobile Phone Number | …….…………………………… |  |  |
| 05 | What is your ethnic group? | Hausa ………………………………….  Igbo …………………………………...  Yoruba ………………………………..  Others (Specify).……………………… | 1  2  3  4 |  |
| 06 | What’s your current marital status? | Single………………………………….  Married………………………………..  Co-habiting…………………………… | 1  2  3 |  |
| 07 | Level of education | No formal Education…………………  Primary……………………………….  Secondary……………………………  Tertiary……………………………… | 1  2  3  4 |  |
| 08 | Where do you live? (specify) | Rural……………………………………  Urban…………………………………… | 1  2 |  |
| 09 | What kind of house structure do you stay in? | Grass thatched/Made of mud…………  Semi-permanent……………………….  Permanent……………………………… | 1  2  3 |  |
| 10 | What sleeping arrangement do you have in place with your under five children? | On same bed with parent………….  On different bed but in the same room ...  In different rooms………………………  No formal sleeping arrangement …… | 1  2  3  4 |  |
| 11 | How many rooms does your house have? | …….…………………………… |  |  |
| 12 | How many people live in your household? | …….…………………………. |  |  |
| 13 | What is your religion? | Christianity……………………………  Islam…………………………………..  African Traditional Religion…………  Others (specify)……………………… | 1  2  3  4 |  |
| 14 | What is your current occupation/job | …….…………………………… |  |  |
| 15 | Husband/Supporter occupation/job | …….…………………………… |  |  |
| 16 | Gravidity | First Pregnancy………………………….  Second Pregnancy………………………  Third Pregnancy and above……………. | 1  2  3 |  |
| 17 | How many children do you have and their ages? | …….……………………………  …….……………………………  …….……………………………  …….……………………………  …….……………………………  …….…………………………… | 1  2  3  4  5 |  |
| 18 | Gestational Age (Months) | …….…………………………… |  |  |
| 19 | What is your average monthly income? (from all sources) | …………………….. |  |  |

**SECTION B: USE OF LONG LASTING INSECTICIDE TREATED NETS (LLINs) AND KNOWLEDGE OF MALARIA**

| **The following questions are to determine the level of use of LLINs and knowledge of malaria** | | | | **Yes** | **No** |
| --- | --- | --- | --- | --- | --- |
| 20 | What do you normally do to prevent mosquito bite? (Tick as many as applicable) | Close doors/windows before nightfall | 1 |  |  |
|  |  | Spray the rooms with insecticide | 2 |  |  |
|  |  | Light coils/insect repellant at night | 3 |  |  |
|  |  | Screen doors/windows with net | 4 |  |  |
|  |  | Use LLIN | 5 |  |  |
|  |  | Do nothing | 6 |  |  |
|  |  | Others(specify) | 7 |  |  |
| 21 | Do you have a Long Lasting Insecticide Treated Net (LLIN)? | Yes | 1 |  |  |
|  |  | No | 2 |  |  |
| 22 | If no, your reason for not having a LLIN could be? (Tick as many as applicable) | I do not know about it | 1 |  |  |
|  |  | I cannot afford the price | 2 |  |  |
|  |  | The design of my bed does not fit | 3 |  |  |
|  |  | I do not think i need it | 4 |  |  |
|  |  | Don’t know where to get one | 5 |  |  |
|  |  | Don’t know the importance | 6 |  |  |
|  |  | My doors/windows are already fitted with net | 7 |  |  |
|  |  | Others (Specify) | 8 |  |  |
| 23 | Have you ever slept under a LLIN? | Never | 0 |  |  |
|  |  | Used to | 1 |  |  |
|  |  | Occasionally | 2 |  |  |
|  |  | Regularly | 3 |  |  |
| 24 | When did you sleep under LLIN in the last 1 week? | Every night | 1 |  |  |
|  |  | Last night | 2 |  |  |
|  |  | 2 nights ago | 3 |  |  |
|  |  | 3 nights ago | 4 |  |  |
|  |  | 4 nights ago | 5 |  |  |
|  |  | 5 nights ago | 6 |  |  |
|  |  | 6 nights ago | 7 |  |  |
|  |  | Not used last week | 8 |  |  |
| 25 | What season of the year do you sleep under LLIN more regularly? | Harmattan/Dry season | 1 |  |  |
|  |  | Raining season | 2 |  |  |
|  |  | Both | 3 |  |  |
| 26 | How does your husband influence the use of LLIN? | Support and encourages the use of the net | 1 |  |  |
|  |  | Does not like the use of the net | 2 |  |  |
|  |  | No influence | 3 |  |  |
|  |  | Others (Specify) | 4 |  |  |
| 27 | Why have you never/occasionally slept under a LLIN? (Tick as many as applicable) | Heat | 1 |  |  |
|  |  | Rashes | 2 |  |  |
|  |  | It does not allow air to circulate freely | 3 |  |  |
|  |  | Never thought of it | 4 |  |  |
|  |  | It’s isn’t our culture | 5 |  |  |
|  |  | Cannot afford it | 6 |  |  |
|  |  | It makes someone shut-in | 7 |  |  |
|  |  | Forgetfulness | 8 |  |  |
|  |  | It irritates the nose | 9 |  |  |
|  |  | It causes bad dreams | 10 |  |  |
|  |  | It gives bad odour | 11 |  |  |
|  |  | Prevent free movement on the bed | 12 |  |  |
|  |  | Problem with permanent space | 13 |  |  |
|  |  | It is used in the hospital only | 14 |  |  |
|  |  | Don’t know where to get it | 15 |  |  |
|  |  | Do not consider it important | 16 |  |  |
|  |  | Cannot tuck it under mat | 17 |  |  |
|  |  | Use other insecticide | 18 |  |  |
|  |  | Malaria fever is only seasonal | 19 |  |  |
|  |  | Mosquitoes still enter them anyway | 20 |  |  |
|  |  | Does not protect against malaria | 21 |  |  |
|  |  | Others(specify) | 22 |  |  |
| 28 | Your reasons for stopping use of a LLIN could be? (Tick as many as applicable) | Only used it in school as a student | 1 |  |  |
|  |  | It is too hot | 2 |  |  |
|  |  | Do not consider it important | 3 |  |  |
|  |  | Cannot afford it | 4 |  |  |
|  |  | Not our culture | 5 |  |  |
|  |  | Never thought of it | 6 |  |  |
|  |  | May be dangerous for children to chew on | 7 |  |  |
|  |  | Others(Specify) | 8 |  |  |
| 29 | What condition will make you use LLIN more regularly? (Tick as many as applicable) | If given free of charge | 1 |  |  |
|  |  | If weather is cool enough | 2 |  |  |
|  |  | If proven to be useful | 3 |  |  |
|  |  | If government make it compulsory | 4 |  |  |
|  |  | If cheaper than other products serving same purpose | 5 |  |  |
|  |  | Others(Specify) | 6 |  |  |
| 30 | Reasons for regular use of LLIN? (Tick as many as applicable) | Prevents malaria | 1 |  |  |
|  |  | Habit formed from school | 2 |  |  |
|  |  | Husband insists | 3 |  |  |
|  |  | Others(Specify) | 4 |  |  |
| 31 | What benefit(s) can you attribute to LLIN? (Tick as many as applicable) | It ensures privacy | 1 |  |  |
|  |  | It prevents mosquito bite | 2 |  |  |
|  |  | It decorates the bed | 3 |  |  |
|  |  | It prevents dust falling on sleeper | 4 |  |  |
|  |  | It prevents insects/reptiles falling on sleeper | 5 |  |  |
|  |  | No benefit | 6 |  |  |
|  |  | Others (Specify) | 7 |  |  |

**KNOWLEDGE OF MALARIA**

| **The following questions examine knowledge of malaria transmission, treatment and prevention** | | | | **Yes** | **No** |
| --- | --- | --- | --- | --- | --- |
| 32 | Possible causes of malaria? (Tick as many as applicable) | Cold weather | 1 |  |  |
|  |  | Excessive sunlight | 2 |  |  |
|  |  | Playing in the rain | 3 |  |  |
|  |  | Eating of bad food | 4 |  |  |
|  |  | When you do not sleep under net | 5 |  |  |
|  |  | From dirty environment | 6 |  |  |
|  |  | Mosquitoes | 7 |  |  |
|  |  | Cold food | 8 |  |  |
|  |  | Beer | 9 |  |  |
|  |  | Stagnant water | 10 |  |  |
|  |  | Others(Specify) | 11 |  |  |
| 33 | Do you think malaria can kill, if it is untreated? | Yes | 1 |  |  |
|  |  | No | 2 |  |  |
| 34 | Groups at high risk of malaria? (Tick as many as applicable) | People living with HIV/AIDS | 1 |  |  |
|  |  | Children under 5 years | 2 |  |  |
|  |  | Pregnant women | 3 |  |  |
|  |  | Everybody | 4 |  |  |
|  |  | Adults | 5 |  |  |
|  |  | Women | 6 |  |  |
|  |  | Others(Specify) | 7 |  |  |
| 35 | Malaria consequences during pregnancy are? (Tick as many as applicable) | Giving birth to a low weight baby | 1 |  |  |
|  |  | Causes mother’s death | 2 |  |  |
|  |  | Foetal death | 3 |  |  |
|  |  | Premature delivery | 4 |  |  |
|  |  | Causes anaemia | 5 |  |  |
|  |  | Others(Specify) | 6 |  |  |
| 36 | What do you think are the most common signs and symptoms of malaria infection? (Tick as many as applicable) | Headache | 1 |  |  |
|  |  | Fever | 2 |  |  |
|  |  | Chills/shivering | 3 |  |  |
|  |  | Vomitting | 4 |  |  |
|  |  | Diarrhoea | 5 |  |  |
|  |  | Body pain | 6 |  |  |
|  |  | Jaundice | 7 |  |  |
|  |  | Loss of energy | 8 |  |  |
|  |  | Loss of appetite | 9 |  |  |
|  |  | Dizziness | 10 |  |  |
|  |  | Joint pain | 11 |  |  |
|  |  | Convulsion | 12 |  |  |
|  |  | Yellow coloured urine | 13 |  |  |
|  |  | Bitterness in mouth | 14 |  |  |
|  |  | Cough | 15 |  |  |
|  |  | Others(Specify) | 16 |  |  |
| 37 | Where do you seek treatment for malaria fever? (Tick as many as applicable) | Herbal home | 1 |  |  |
|  |  | Chemist | 2 |  |  |
|  |  | Hospital/Clinic/Health centre | 3 |  |  |
|  |  | Prayer house | 4 |  |  |
|  |  | No where | 5 |  |  |
|  |  | Others(Specify) | 6 |  |  |
| 38 | Do you think malaria can be prevented? | Yes | 1 |  |  |
|  |  | No | 2 |  |  |
| 39 | Malaria prevention practices include? (Tick as many as applicable) | Sleep under mosquito net | 1 |  |  |
|  |  | Sleep under LLINs | 2 |  |  |
|  |  | Maintaining clean environment | 3 |  |  |
|  |  | Indoor spraying | 4 |  |  |
|  |  | Use of window and door nets | 5 |  |  |
|  |  | Eliminate stagnant water around living area | 6 |  |  |
|  |  | Use of drugs | 7 |  |  |
|  |  | Cut the grass | 8 |  |  |
|  |  | Use of traditional herbs | 9 |  |  |
|  |  | Keep doors and windows closed | 10 |  |  |
|  |  | Use of mosquito coil | 11 |  |  |
|  |  | Use of insect repellent | 12 |  |  |
| 40 | What can a pregnant woman do to prevent malaria? (Tick as many as applicable) | Sleep under mosquito net | 1 |  |  |
|  |  | Sleep under LLIN | 2 |  |  |
|  |  | Keep environment clean | 3 |  |  |
|  |  | Take SP/Fansidar given during antenatal care | 4 |  |  |
|  |  | Take Daraprim tablets (Sunday-Sunday medicine) | 5 |  |  |
|  |  | Others(Specify) | 6 |  |  |
| 41 | Can malaria be treated? | Yes | 1 |  |  |
|  |  | No | 2 |  |  |
| 42 | What drugs are used to treat adults with malaria? (Tick as many as applicable) | SP/Fansidar | 1 |  |  |
|  |  | Chloroquine | 2 |  |  |
|  |  | Quinine | 3 |  |  |
|  |  | ACT | 4 |  |  |
|  |  | Aspirin, panadol, paracetamol | 5 |  |  |
|  |  | Others(Specify) | 6 |  |  |
| 43 | What drugs are used to treat children with malaria? (Tick as many as applicable) | SP/Fansidar | 1 |  |  |
|  |  | Chloroquine | 2 |  |  |
|  |  | Quinine | 3 |  |  |
|  |  | ACT | 4 |  |  |
|  |  | Panadol, paracetamol | 5 |  |  |
|  |  | Others(Specify) | 6 |  |  |

**SECTION C: Malaria Microscopy Results**

**0:- Negative;**

**1+ :- 1 – 10 parasites per 100 High Power Field (HPF)**

**2+ :- 11 – 100 parasites per 100 HPF**

**3+ :- 1 – 10 parasites per HPF**

**4+ :- 11 – 100 or > 10 parasites per HPF**

| **Microscopy Result** | Initial | 12 Weeks |
| --- | --- | --- |
| Level of parasitaemia |  |  |
